# Minimal assessment of the index case: the point of view of the cardiologist

**DOI:** 10.1186/1750-1172-10-S1-I21

**Published:** 2015-11-02

**Authors:** Michel Slama

**Affiliations:** 1 Hôpital Antoine-Béclère, Clamart, France

## 

TTR familial amyloidosis is a systemic disease, with a frequent cardiac involvement

For clinical reasons – related to its prolonged latency - and historical reasons – as the first cases where diagnosed by neurologists, in young patients who died from neurological disease before cardiac complications occurred- familial cardiac amyloidosis (TTR-FAC) has been and still remains underestimated. It is known to be responsible of 40% of mid-term deaths after liver transplantation. TTR-FAC appears irreversible; it can progress after liver transplantation, and is associated with a poor prognosis. Hence the heart deserves a special attention when taking in charge patients with familial amyloidosis, especially since there are several new promising therapeutic options.

When following patients with a TTR mutation, the occurrence of a cardiac disease can be anticipated considering gene mutation (opposing the so called cardiac vs neurologic mutations), family history, and age, but as it can be observed in all types of mutations, with various frequencies, it can never be ruled out beforehand. The initial workup of those patients should take into account:

- Cardiac infiltration, responsible for restrictive cardiomyopathy, detected by clinical examination, assessment of cardiac capacity with a 6 mn walk test, ECG (microvoltage), echocardiography with typical thickened sparkling myocardium, decrease of longitudinal systolic deformation (strain), and biomarkers (NT pro BNP, troponin). Early detection of amyloid deposits can be obtained by MRI (T1 mapping) and bisphosphonate scintigraphy. Cardiac catheterization and myocardial biopsy are rarely mandatory.

- Conduction abnormalities can be responsible of syncope by atrioventricular block. A simple ECG assessment is a good start, and any abnormality (any degree of AV block, bundle branch block) should prompt electrophysiological study. Prophylactic pacemaker implantation is widely used; in our experience it is necessary in about 1/3 of patients during the course of the disease. Malignant arrhythmias (ventricular tachycardia or fibrillation) appear uncommon.

- Cardiac sympathetic and parasympathetic denervation, as assessed by cardiac variability (Holter), atropine challenge, and MIBG scintigraphic imaging, occurs very early, and has a very strong prognostic value.

The initial cardiac assessment of patients with TTR amyloidosis should not be limited to the evaluation of obvious cardiac damage, which is irreversible and can lead to cardiac transplantation in a limited number of patients. All our efforts must be directed to achieve a very early diagnosis of TTR-FAC, which should be taken into account to start the treatment as early as possible.

In the era of SiRNA therapy for this rare and deadly disease, the “minimal” cardiac assessment should be performed in specialized reference centers, devoting their efforts to research for very early and accurate diagnosis and development of new therapies, and should by no means be minimal.

